# Understanding natural killer cell regulation by mathematical approaches

**DOI:** 10.3389/fimmu.2012.00359

**Published:** 2012-12-12

**Authors:** Carsten Watzl, Michal Sternberg-Simon, Doris Urlaub, Ramit Mehr

**Affiliations:** ^1^IfADo - Leibniz Institute for Occupational ResearchDortmund, Germany; ^2^The Mina and Everard Goodman Faculty of Life Sciences, Bar-Ilan UniversityRamat Gan, Israel

**Keywords:** natural killer cells, mathematical modeling, systems biology, signaling pathways, activating receptors, inhibitory receptors, proliferation

## Abstract

The activity of natural killer (NK) cells is regulated by various processes including education/licensing, priming, integration of positive and negative signals through an array of activating and inhibitory receptors, and the development of memory-like functionality. These processes are often very complex due to the large number of different receptors and signaling pathways involved. Understanding these complex mechanisms is therefore a challenge, but is critical for understanding NK cell regulation. Mathematical approaches can facilitate the analysis and understanding of complex systems. Therefore, they may be instrumental for studies in NK cell biology. Here we provide a review of the different mathematical approaches to the analysis of NK cell signal integration, activation, proliferation, and the acquisition of inhibitory receptors. These studies show how mathematical methods can aid the analysis of NK cell regulation.

## INTRODUCTION

Natural killer (NK) cells are innate lymphocytes that are important for early immune responses against viral pathogens and transformed cells (Vivier et al., 2008). They can be stimulated by cytokines or by direct contact with infected or transformed cells, resulting in cytolysis of the recognized cell and the production of immune-modulatory cytokines. Target cell recognition is mediated by an array of stimulatory and inhibitory NK cell receptors (Lanier, 2008). The opposing signals induced by these receptors regulate NK cell activities (Watzl and Long, 2010). Inhibitory receptors are necessary to ensure NK cell self-tolerance in two ways. During NK cell development inhibitory receptors specific for self-MHC class I are necessary for the generation of functional competent NK cells in a process termed “education” (Elliott and Yokoyama, 2011). In mature NK cells the presence of inhibitory receptors prevents the attack of healthy autologous cells (Kärre,, 2008). To study the acquisition of inhibitory receptors, their exact role during NK cell education and their interference with activating signaling is essential for the understanding of NK cell regulation.

The immune system is one of the most complex biological systems known. It involves various cell types including lymphocytes, each of which can exhibit a diverse repertoire of individual cells: T and B cells express clonally distributed somatically rearranged antigen receptors, whereas each NK cell can express different inhibitory and activating receptors. These cells do not act as individuals, but interact with other immune cells in a complex inter-cellular network. Furthermore, lymphocyte activation is regulated by a complicated intra-cellular signaling machinery. As a result, the immune system and its functionality are highly complex and non-linear. The analysis of immune system data is thus non-trivial and calls for the use of systems biology methods, including mathematical and computational models (Walzer et al., 2007).

## MATHEMATICAL METHODS TO ANALYZE AND MODEL IMMUNE SYSTEM DATA

Obtaining, analyzing, and comparing data on lymphocyte repertoires yields many new insights (reviewed in Mehr et al., 2012). However, the formidable challenge is to formulate a description of the system in question on several levels: genetic, molecular, cellular, and systemic. Of course, no model can include all aspects of the real system – nor should it attempt to, because models must be simple enough to enable an exact analysis. It is up to the researcher to decide which aspects of the system to focus on, and which aspects to neglect (Mehr, 2001, 2005).

Before performing any multi-scale modeling, one should know how to model events on a single scale. These methods can be combined in multi-scale modeling in various ways, using for each scale the most appropriate method (Shannon and Mehr, 1999; Kirschner et al., 2007; Materi and Wishart, 2007). The main modeling techniques used so far in immunology are: models of population dynamics using ordinary differential equations (ODEs), including delay differential equations (DDE), or – in case the numbers of objects (e.g., cells or molecules) is not very large and stochastic effects are important – stochastic differential equations (SDEs). The latter (and other so-called “Monte Carlo” modeling methods, such as stochastic agent-based models – see below) have been developed to better account for intrinsic cellular and molecular fluctuations. For example, see our (mostly ODE) models of lymphocyte development – T cell development in the thymus (Mehr et al., 1993, 1994, 1995, 1996a,b, 1997, 1998) and homeostasis in peripheral blood (Mehr and Perelson, 1997), and ODE or hybrid models of B cell development and responses (Mehr et al., 2003; Shahaf et al., 2004, 2006, 2010). Stochastic modeling has also been extensively used by several groups, e.g., the Hodgkin group’s “Cyton” model (Lee et al., 2009; Markham et al., 2010; Duffy and Hodgkin, 2012; Duffy et al., 2012). In case spatial effects are important, one can either divide the space into compartments and still use ODE modeling for each compartment, or use partial differential equations (PDE), which explicitly model the dependence of the model’s entity on space. A different method which has become popular with the availability of high-speed computation, and is also more intuitive and accessible for biologists, is agent-based modeling (ABM) – where individual entities (agents) such as molecules, cells or individuals are explicitly modeled as program objects, which move, respond to their environment and/or change their internal state based on pre-defined rules (Chavali et al., 2008). Stochasticity is easy to build into ABM, as are different levels of description. One can also create hybrid models where different levels of description are modeled using different model types (Shahaf et al., 2008). Many such models of lymphocyte migration or molecular processed were published (e.g., Beltman et al., 2011; Kaplan et al., 2011). A review of ODE, DDE, PDE, stochastic, and ABM models in immunology, including a comparison of their relative advantages and disadvantages, has recently been published (Kim et al., 2009).

## MODELING NK CELL SIGNALING PATHWAYS AND THE DYNAMICS OF NK CELL ACTIVATION

The understanding of T cell receptor signaling has been greatly aided by mathematical approaches (for examples, see Feinerman et al., 2008; Das et al., 2009). NK cells rely on similar signaling pathways for their activation. However, NK cell activation is mediated by several different activating receptors, which can synergize with each other (Bryceson et al., 2006). Additionally, inhibitory receptors can counteract NK cell activation (Watzl and Long, 2010). This makes NK cell regulation even more complex. Analyzing and predicting the behavior of NK cell repertoires necessitates models that take into account the complexity of whole NK cell repertoires (Johansson et al., 2009), as well as the functional differences between NK cell repertoire subsets (Brodin et al., 2010, 2012) and the molecular processes underlying these differences. Mathematical modeling may therefore help to understand the integration of these opposing signals. However, the lack of detailed knowledge about the complex topology of NK cell signaling pathways presents a challenge for modeling approaches.

In a purely theoretical approach using stochastic agent-based simulation one study has developed a detailed molecular model of NK cell activation, incorporating membrane-proximal signals and affinities of receptor–ligand interactions (Das, 2010). The engagement of activating receptors results in the activation of the Vav and Erk signaling pathways. Interestingly, the study showed that on a single cell level the responses can be digital in nature so that the latter signaling pathways are either active or not. Such digital behavior of Erk phosphorylation matches previous results in T cells (Altan-Bonnet and Germain, 2005), where opposing feedback loops of positive (Erk) and negative (SHP-1) signals created a digital response. However, the model showed that in NK cells such a digital response is possible even in the absence of any feedback loop (Das, 2010), because of the presence of inhibitory receptors in the NK cell synapse.

Another study used an ODE-based “ensemble modeling” approach (Kuepfer et al., 2007) combined with experimental verification to investigate the interplay of activating and inhibitory signals in NK cells (Mesecke et al., 2011). This study experimentally confirmed a digital Vav phosphorylation and de-phosphorylation induced by activating and inhibitory signaling, respectively. The latter result suggests that Vav phosphorylation is an integration point at which activating and inhibitory signaling converge (Stebbins et al., 2003). The digital nature of Vav phosphorylation (Das, 2010; Mesecke et al., 2011) could explain how these opposing signals are integrated to yield a yes or no decision about NK cell activity, resulting in the survival or the death of a conjugated target cell.

The inhibition of NK cells depends on the amount of MHC class I expressed by the target cell. It has been shown that there is a clear threshold in the amount of surface MHC class I required for inhibition of NK cell cytotoxicity. A study by Almeida et al. modeled the dependence of the activation/inhibition threshold on MHC class I and inhibitory receptor expression levels at the single cell level using ODEs (Almeida et al., 2011). They fitted their model to data on NK cell killing of target cells with various MHC levels, obtained in experiments from many different human NK cell clones, in order to see whether clones differ in their activation threshold, maximal killing capacity, or both. The results showed that NK cells differ mostly in their intrinsic activation threshold, which may be set during the process of NK cell education.

An agent-based modeling study by Kaplan et al. (2011) addressed the question how signals from activating and inhibitory receptors are spatially integrated within the NK cell immunological synapse. The study constructed a computer simulation of the assembly of the NK cell-target cell synapse, which was based on many experimental findings. The NK cell immune synapse was modeled as two parallel two-dimensional grids, representing the contact areas on the membranes of the two interacting cells – the NK and the target cell grids. The simulation suggested that the NK cell does not simply sum up all activating and inhibitory signals over the entire synapse. Instead, a model considering that the inhibitory signal is locally confined to a radius of 3–10 receptor molecules was closer to the experimentally observed behavior. This is in line with the fact that inhibitory receptors mediate their negative signal through direct recruitment of a protein tyrosine phosphatase such as SHP-1, and suggests that the active phosphatase cannot diffuse very fast. Therefore, inhibitory receptors have a locally limited inhibition radius.

The simulation by Kaplan et al. (2011) further showed that the concerted motion of receptors in clusters – such as those formed by molecule aggregation on certain membrane microdomains – significantly accelerates the maturation of the NK cell immunological synapse. Membrane microdomains have also been implicated by the study of Mesecke et al. The mathematical modeling showed that the association of Src-family kinases with activating but not with inhibitory receptors was essential for the experimentally observed Vav activation (Mesecke et al., 2011). While this might suggest a direct association between activating NK cell receptors and Src-family kinases, it could also support the known role of membrane microdomains in NK cell activation (Lou et al., 2000; Fassett et al., 2001; Watzl and Long, 2003; Endt et al., 2007; Guia et al., 2011). As these domains are enriched in Src-family kinases, the recruitment of activating receptors to these domains might be the event that brings kinases and receptors in close proximity. Additionally, clustering of activating receptors would make them more resistant to the locally restricted negative signal of inhibitory receptors (Kaplan et al., 2011).

A modeling study by Burroughs et al. (2011) addressed the question of receptor segregation at the NK cell immunological synapse. They specifically asked the question of whether the segregation of different receptors at the immunological synapse is simply a result of the different sizes of the proteins. They used a novel energy model that can analyze protein redistribution and developed a methodology for analyzing and quantifying data from two color fluorescence images. Using these methods, they showed that the observed protein segregation patterns in actual synapses can be explained by differences in protein size alone. This was independent of the actin cytoskeleton and could explain how certain receptor patterns form within the immunological synapse.

Once a NK cell has been sufficiently stimulated by a locally attached target cell, a cytotoxic process is initiated, resulting in the lysis of the target cell. A study by Almeida et al. (2011), explored the kinetics of NK cell activation and target cell lysis at the population level. Experiments have shown that while the number of NK cell-target cell conjugates reaches saturation within 30 min, significant target cell lysis is only observed after 3–5 h. A model assuming that the NK cell can kill its target cell only after being primed by a former target cell encounter could reproduce the experimentally observed kinetics and explain this delay. Thus, the population of NK cells is divided into resting NK cells that do not kill targets, and activated NK cells that are capable of killing. This suggests that NK cells need to be primed before they can kill target cells, and that this priming occurs after the first encounter of the resting NK cell with a target cell.

## NK CELL POPULATION DYNAMICS

Mathematical modeling has been used in several studies to elucidate the dynamics of NK cell populations, their development and activation. Early mechanistic modeling studies of NK cell population dynamics (as opposed to simple curve-fitting studies of killing dynamics) included attempts to clarify their role in immune surveillance (Merrill, 1981, 1983) and in immunotherapy (Nani and Oguztoreli, 1994), or elucidate the dynamics of their adhesion to target cells (Kuznetsov, 1996). In recent years, there have been several more mathematical modeling studies on the role of NK cells in response to infections (Hancioglu et al., 2007; Wodarz et al., 2007; Elemans et al., 2011) and cancers (Cappuccio et al., 2006). In many of these studies, however, the models focus on the adaptive T cell response, and NK cells are just an additional feature. We are only aware of one study, which focused primarily on NK cell population dynamics and turnover (Zhang et al., 2007). Recently, one report investigated the proliferation of NK cells in response to IL-15 (Zhao and French, 2012). IL-15 plays an important role in NK cell development, survival and homeostasis, but also during virus-induced NK cell proliferation. The authors developed a series of two-compartment models describing the dividing and non-dividing NK cell subpopulations. NK cells could be recruited from one compartment into the other and possessed distinct proliferation and cell death rates within a compartment. Comparing experimental results to different model variations showed that IL-15 modulates the recruitment rate from the non-dividing to the dividing compartment and influences the division rates. However, it did not have any influence on the time until the first division. The model could even predict NK cell proliferation dynamics in an environment of changing IL-15 concentrations, as it would be the case during viral infections, demonstrating the usefulness of such mathematical models.

## NK CELL REPERTOIRE FORMATION

The dynamics of NK cell repertoire development and education are even more complex than the population dynamics of NK cell subsets. Initially, two conceptual models have been proposed by Vance and Raulet (1998), to explain the process of NK cell “education” in which the cells adapt to the self-MHC environment. Both models have assumed that in order to mature, there should be at least one self-MHC-specific receptor on the NK cell (the “at least one” hypothesis). The first is the sequential activation model. In this scheme, genes for Ly49 inhibitory NK cell receptors are randomly expressed; once a receptor gene is expressed, it is known to remain stably activated. During maturation, the cell expresses new receptors, until a receptor that binds self-MHC is expressed. The cells are periodically tested for interaction with self-MHC molecules. Strong interactions between the receptors and their MHC ligands prevent additional receptors from being expressed and result in the maturation of the cell. In this scheme, a single testing step accomplishes the education process; however this single step may be repeated many times during the cell’s development. The second scheme is the two-step selection model, in which the repertoire is fully formed at an initial stage by a stochastic process of receptor expression, and subsequently shaped by two selection steps: one selects for cells expressing at least one self-specific receptor, and the other selects against cells expressing multiple self-specific receptors. The two-step selection thus occurs only once for each cell, when it has completed its receptor gene activation. Depending on the signaling thresholds at these steps, the process may allow maturation of cells expressing more than one self-specific receptor.

Mathematical methods are ideally suited to distinguish between these two models. In the study by Salmon-Divon et al. (2003a), the “two-step” model was implemented as a computer program which calculates the expected frequencies of cells with given receptor expression patterns as function of the selection thresholds. The “sequential” model of NK cell selection was implemented as a stochastic agent-based simulation of the development of the NK cell repertoire, again giving the expected frequencies of cells with given receptor expression patterns based on model parameters. The original Raulet group data was not sufficient to conclusively decide between the models (Salmon-Divon et al., 2003b). However, the use of specifically generated, larger data set from single-MHC mice showed that the two-step selection model fits the data significantly better than the sequential model (Johansson et al., 2009).

Additional studies aimed to determine whether the MHC class I background affects NK cell repertoire composition, in both humans and mice. For this purpose, data on NK cell repertoires extracted from the bone marrow (BM) of mice from different single-MHC and MHC-deficient backgrounds was analyzed. In addition, NK cell repertoire data from blood samples of humans with different MHC backgrounds was used. In both cases, cells were probed for the expression of five inhibitory receptors (from the Ly49 or KIR families, in mice and humans, respectively). The results of these studies are described in another paper in this issue (Simon, M. et al., submitted).

## OUTLOOK

There are still many challenging and open questions about different aspects of NK cell regulation to date. We still need to learn more about NK cell education, subsets, signaling and even memory (Paust and von Andrian, 2011). The studies described above have demonstrated that mathematical approaches can help to answer some of these open questions. They are ideally suited to analyze complex data sets, make predictions about complicated systems or simply aid experimental design. We therefore expect to see many more studies involving mathematical approaches to NK cell regulation in the near future.

## Conflict of Interest Statement

The authors declare that the research was conducted in the absence of any commercial or financial relationships that could be construed as a potential conflict of interest.

## Acknowledgments

The studies in the Mehr lab that are described in this review have benefited from our collaborations with Catarina R. Almeida, Petter Brodin, Daniel M. Davis, Marjet Elemans, Petter Höglund, Maria Johansson, Sofia Johansson, Klas Kärre, Karl-Johan Malmberg, Peter Parham, Markus Uhrberg, and Eric Vivier. We are indebted to all of them for the data and fruitful discussions that led to the work described herein. These studies were funded over the years by The Israel Science Foundation (grant number 759/01 and Bikura grant number 1503/08); The Human Frontiers Science Program – a Young Investigator Grant; and a Systems Biology Prize grant from Teva Pharmaceuticals. The collaborative work on NK cells with groups in Sweden was supported by the Swedish Foundation for Strategic Research and the Swedish Research Council. The studies from the Watzl lab were supported by grants from the Deutsche Forschungsgemeinschaft (TRR83-TP16, WA 1552/2-1), the BMBF (BioFuture grant and the Helmholtz-Alliance on Systems Biology, SBCancer) and the Deutsche Krebshilfe.
